# Passive Immunity to *Vibrio cholerae* O1 Afforded by a Human Monoclonal IgA1 Antibody Expressed in Milk

**DOI:** 10.20411/pai.v5i1.370

**Published:** 2020-05-08

**Authors:** Danielle E. Baranova, Lihow Chen, Margaret Destrempes, Harry Meade, Nicholas J. Mantis

**Affiliations:** 1 Department of Biomedical Sciences; University at Albany; Albany, New York; 2 Division of Infectious Diseases; Wadsworth Center; New York State Department of Health; Albany, New York; 3 LFB USA; Framingham, Massachusetts

**Keywords:** enteric, immunity, cholera, antibody, vaccine, mucosal, milk

## Abstract

**Background:**

In cholera epidemics, the spread of disease can easily outpace vaccine control measures. The advent of technologies enabling the expression of recombinant proteins, including antibodies, in the milk of transgenic animals raises the prospect of developing a self-administered and cost-effective monoclonal antibody (MAb)-based prophylactic to reduce the incidence of *Vibrio cholerae* infection.

**Methods:**

We generated a transgenic mouse line in which the heavy and light chain variable regions (Fv) specific for a conserved epitope in the core/lipid A of *V. cholerae* O1 lipopolysaccharide were expressed as a full-length human dimeric IgA1 (ZAC-3) and secreted into the milk of lactating dams. Milk containing ZAC-3 IgA1 was assessed for the ability to passively protect against experimental cholera infection in a newborn mouse model and to impact bacterial swimming behavior.

**Results:**

Newborn mice that were passively administered ZAC-3 IgA1 containing milk, or that suckled on dams expressing ZAC-3 IgA1, were immune to experimental cholera infection, as measured by a reduction of *V. cholerae* O1 colony forming units recovered from intestinal lysates 12 hours after oral challenge. *In vitro* analysis revealed that ZAC-3 hIgA1-containing milk arrested *V. cholerae* motility in soft agar and liquid media and was effective at promoting bacterial agglutination, possibly accounting for the observed reduction in bacterial colonization *in vivo*.

**Conclusions:**

These results demonstrate that consumption of milk-derived antibodies may serve as a strategy to passively protect against cholera and possibly other enteric pathogens.

## INTRODUCTION

Cholera is a severe diarrheal disease that can cause death within hours in the absence of intravenous rehydration therapy [1]. The disease is distributed globally with the highest incidence occurring in association with regional outbreaks. In the past 3 years in Yemen alone, there have been more than 2 million cases of cholera and more than 3,800 deaths [2]. The etiological agent of this disease is the Gram-negative bacterium, *Vibrio cholerae*. The bacterium is transmitted through the consumption of contaminated water and food, with the spread of disease exacerbated by breakdowns in municipal infrastructures due to natural disasters or civil conflicts. Upon entering the digestive tract, *V. cholerae* utilizes a single polar flagellum to reach the proximal small intestine, where it penetrates the viscous mucus layer overlying the epithelium, and gains access to villus crypts. Interaction with the intestinal epithelium is mediated by the toxin-coregulated pilus (TCP). Following intestinal colonization, the bacterium secretes cholera toxin (CT), an ADP-ribosylating toxin that triggers severe watery diarrhea (rice water stool) that is the hallmark of the disease and the main driver of mortality [3].

*V. cholerae* species are divided into over 200 serogroups defined by their O-polysaccharide (OPS) antigen, although only the O1 and O139 serogroups are known to cause epidemics. Since 1817 there have been 7 cholera pandemics. The first 6 pandemics were attributed to the classical bio-type of *V. cholerae* O1, while the current pandemic is caused predominantly by the El Tor biotype [4, 5]. The *V. cholerae* O1 classical and El Tor biotypes differ in several important respects, including the ability of most El Tor isolates to outcompete classical isolates both *in vitro* and *in vivo*. El Tor strains also have an increased ability to transition between highly motile, planktonic forms and non-motile, biofilm states, leading to increased fitness in marine environments [6–8], Additionally, due to differences in the source of bacteriophage encoding CT and the regulation of key virulence genes, classical strains secrete more CT and cause more severe disease in humans [1, 6, 9]. Interestingly, there are circulating variant El Tor strains which possess the CT genetic element from classical strains, and they cause more severe disease than their predecessor seventh pandemic El Tor strains, which is thought to be due to this difference in CT genetic element source [10].

Individuals who experience an episode of cholera develop serotype-specific IgG and IgA antibodies in serum and intestinal secretions, respectively. The bulk of the human antibody response is directed against 2 targets: CT and the OPS of lipopolysaccharide (LPS) [11]. In terms of immunity, protection is associated with anti-OPS antibodies, not anti-CT antibodies [12–17]. Anti-CT serum and mucosal antibodies are apparently ineffective at protecting against disease because the toxin is released from the bacterium directly onto the epithelium, with little opportunity for antibodies to interfere with toxin binding or uptake [14]. Anti-LPS IgA antibodies, on the other hand, interfere with the earliest steps in *V. cholerae* infection. Specifically, antibodies against OPS or the core/lipid A region of LPS (see below) have been shown to arrest *V. cholerae* motility and promote bacterial agglutination [18–25]. a severe diarrheal disease that remains endemic in many parts of the world and can cause outbreaks wherever sanitation and clean water systems break down. Prevention of disease could be achieved through improved sanitation and clean water provision supported by vaccination. *V. cholerae* serogroup O1 is the major cause of cholera; O1 serotypes Inaba and Ogawa have similar disease burdens, while O139 is the only non-O1 serogroup to cause epidemics. We showed previously that immunization of adult female mice with purified *V. cholerae* outer membrane vesicles (OMVs. In liquid culture, for example, *V. cholerae* stops swimming within minutes of being treated with LPS-specific polyclonal or monoclonal antibodies (MAb). Agglutination is the result of the formation of large macroscopic aggregates that are likely entrapped within intestinal mucus and cleared from the gut through a process known as immune exclusion [26].

Despite the success of oral cholera vaccines (OCV) in regions where cholera is endemic, there remains a need for an effective pre-exposure prophylactic (PrEP) that could be administered to individuals in outbreak situations where vaccine implementation is not immediately possible. One possible avenue is oral passive immunization with polyclonal or monoclonal antibodies. In the case of enterotoxigenic *Escherichia coli* (ETEC), it has been demonstrated in Phase 1 clinical trials that repeated oral delivery of hyperimmune bovine colostrum (HBS) affords protection against experimental traveler's diarrhea [27]. In the case of cholera, there is considerable evidence that anti-LPS IgA antibodies are protective when passively administered to newborn mice in an experimental cholera challenge model [14, 17, 18]. In humans, there is also literature indicating that anti-LPS IgA antibodies in breast milk protect against disease [28]. Thus, platforms such as directed expression of MAb in milk of transgenic animals might be ideal for cholera applications [29].

As a proof of principle, we sought to test whether a recombinant anti-LPS IgA MAb expressed in milk can afford protection against experimental cholera challenge in neonatal mice. The MAb of choice for these studies was ZAC-3 IgA. ZAC-3 targets an epitope within the core/lipid A region of LPS that is conserved among *V. cholerae* O1 classical and El Tor isolates [22, 30, 31]. ZAC-3, as either an IgA or IgG, is a particularly potent inhibitor of *V. cholerae* motility in liquid and semi-solid media. ZAC-3 also promotes *V. cholerae* agglutination and stimulates the bacteria to secrete an extracellular matrix (ECM) reminiscent of the early stages of biofilm formation [32].

In this study, we generated transgenic mice in which ZAC-3 was secreted into mouse mamma-ry glands and secreted into the milk of lactating dams as a full-length human dimeric IgA1. In the newborn mouse model of cholera we show that milk containing ZAC-3 hIgA1 significantly reduced *V. cholerae* O1 colonization of the intestinal mucosa. *In vitro*, milk containing ZAC-3 hIgA1 curtailed *V. cholerae* motility in soft agar and liquid media and was effective at promoting bacterial agglutination, possibly accounting for the observed reduction in bacterial colonization *in vivo*. These results demonstrate that consumption of milk-derived antibodies may serve as a strategy to passively protect against cholera and possibly other enteric pathogens.

## MATERIALS AND METHODS

### Growth conditions for bacterial strains

The bacterial strains used in this study are described in Supplemental Table 1. Bacteria were grown in LB medium at 37°C with aeration, 200 rpm. As necessary, media were supplemented with 100 μg/mL of streptomycin or 10 μg/mL of gentamicin.

### Generation of fluorescent *V. cholerae*

A high copy plasmid was engineered to constitutively express mCherry in *V. cholerae*. To accomplish this, the mCherry open reading frame was first amplified from pMQCherry80 (Matthew Wargo, unpublished) using Q5 DNA polymerase (NEB) and the xfp_ORF_SOE_F2 and mCherry_R_HindIII primer set. A constitutive derivative of the Plac promoter (PA/01/04/03) was similarly amplified from pUC18-mTn7T-eyfp-Gm [33] with the PA1/04/03_ORF_SOE_R1 and PA1/04/03_F_KpnI primer set. The resulting mCherry and PA/01/04/03 fragments were gel extracted using Thermo Scientific GeneJet kit (Cat. No. K0502) and fused together through overlap extension PCR using Q5 DNA polymerase (NEB, Cat. No. M0492S) and the PA1/04/03_F_KpnI forward and mCherry_R_HindIII reverse primers to create PA/01/04/03::mcherry. The fusion product was subsequently digested with KpnI and HindIII (NEB), ligated into similarly cut pUC18T-mTn7T-eyfp (replacing the eyfp ORF and associated promoter) [33], transformed into NEB 5α chemically competent cells, and plated on LB agar supplemented with 10 µg/mL of gentamicin. Plasmid DNA was harvested from the gentamicin-resistant colonies that emerged, using miniprep (Qiagen), and then screened by restriction digest for the presence of the reporter gene. The resulting plasmid, pGW104, was then transformed into *V. cholerae* O395 using electroporation to create DB364. Primer sequences are listed in Supplemental Table 2.

### Monoclonal antibodies and cell lines

Monoclonal recombinant human ZAC-3 IgG1 was used as a reference [34]. Human IgA from colostrum was used a positive control in IgA sandwich ELISAs (RRID:AB_1163623). Goat anti-human IgG-HRP (RRID:AB_228265) and a goat anti-human IgA HRP conjugate (MP Biomedicals) secondary antibodies were utilized in ELISAs.

### Construction and production of ZAC-3 IgA1 HC, LC, and J chain

**Supplementary Figure 1.**
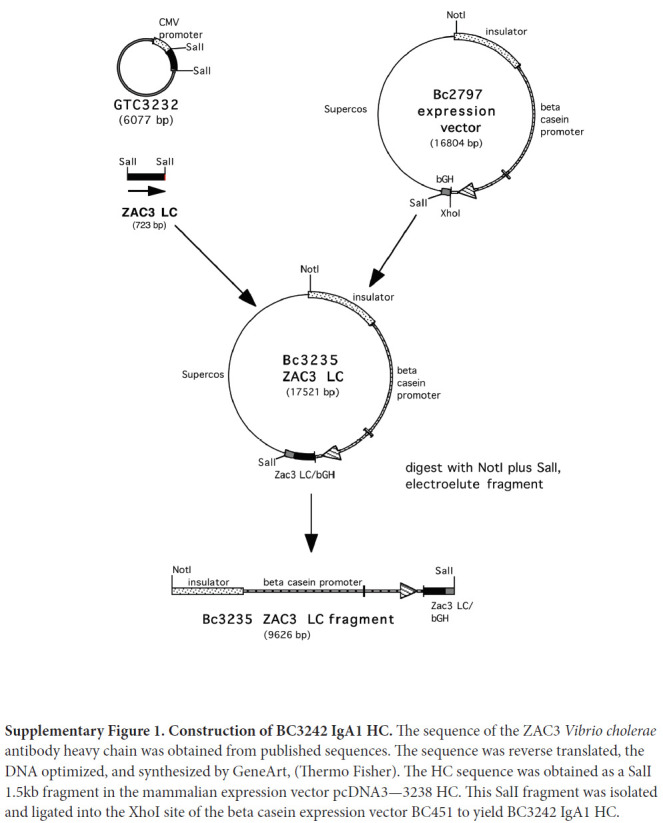
**Construction of BC3242 IgA1 HC.** The sequence of the ZAC3 *Vibrio cholerae* antibody heavy chain was obtained from published sequences. The sequence was reverse translated, the DNA optimized, and synthesized by GeneArt, (Thermo Fisher). The HC sequence was obtained as a SalI 1.5kb fragment in the mammalian expression vector pcDNA3—3238 HC. This SalI fragment was isolated and ligated into the XhoI site of the beta casein expression vector BC451 to yield BC3242 IgA1 HC.

**Supplementary Figure 2.**
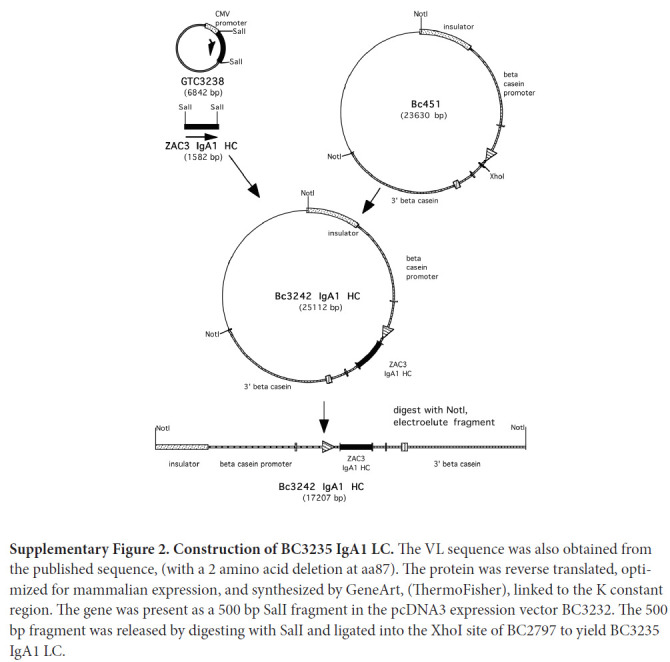
**Construction of BC3235 IgA1 LC.** The VL sequence was also obtained from the published sequence, (with a 2 amino acid deletion at aa87). The protein was reverse translated, optimized for mammalian expression, and synthesized by GeneArt, (ThermoFisher), linked to the K constant region. The gene was present as a 500 bp SalI fragment in the pcDNA3 expression vector BC3232. The 500 bp fragment was released by digesting with SalI and ligated into the XhoI site of BC2797 to yield BC3235 IgA1 LC.

**Supplementary Figure 3.**
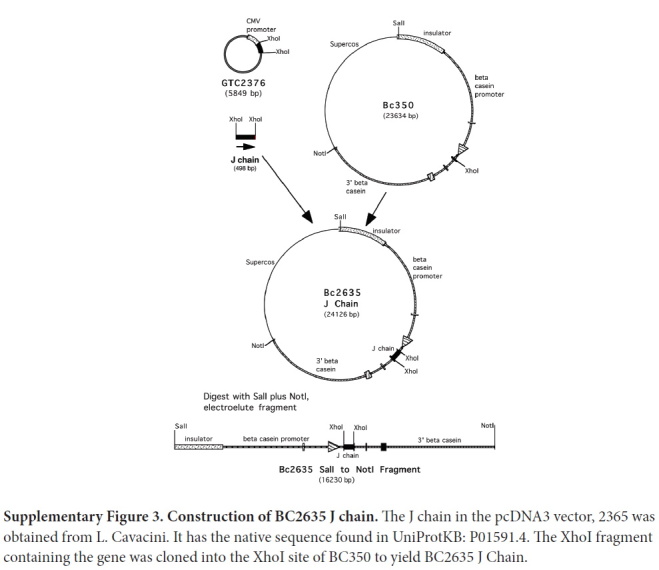
**Construction of BC2635 J chain.** The J chain in the pcDNA3 vector, 2365 was obtained from L. Cavacini. It has the native sequence found in UniProtKB: P01591.4. The XhoI fragment containing the gene was cloned into the XhoI site of BC350 to yield BC2635 J Chain.

**Supplementary Figure 4.**
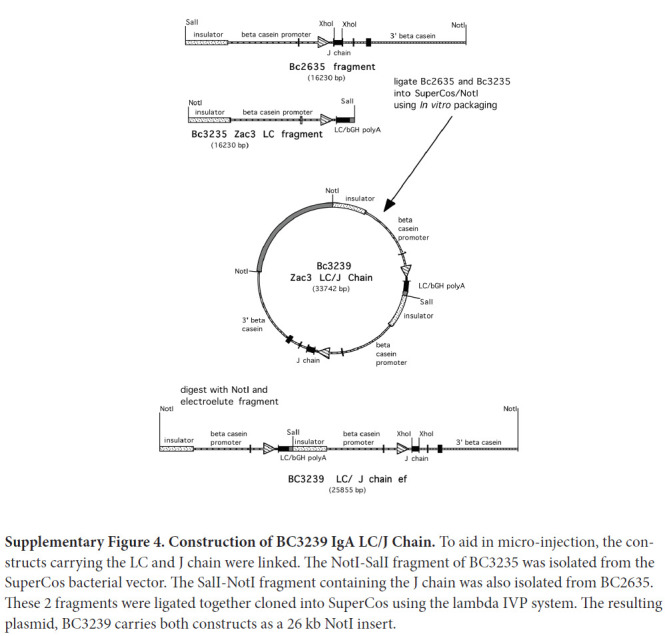
**Construction of BC3239 IgA LC/J Chain.** To aid in micro-injection, the constructs carrying the LC and J chain were linked. The NotI-SalI fragment of BC3235 was isolated from the SuperCos bacterial vector. The SalI-NotI fragment containing the J chain was also isolated from BC2635. These 2 fragments were ligated together cloned into SuperCos using the lambda IVP system. The resulting plasmid, BC3239 carries both constructs as a 26 kb NotI insert.

The beta casein expression vectors contain 6.1kb of upstream promoter sequence linked to an *Xho*I cloning site, located just before the translation start of the beta casein coding sequence. Downstream is either a 7.2 kb downstream sequence including the last 3 exons of beta casein (BC350, BC451) or the 300 bp bgH polyA sequence, (BC2797). There is a 2.4 kb sequence encoding the chicken beta globin hypersensitive site that is positioned upstream of the casein promoter. The amino acid sequence of the ZAC-3 variable regions was taken from a previous publication [34]. The heavy chain (HC) variable region was successfully used to generate HC constructs of IgA1. The variable light chain (LC) sequence was copied and in so doing 2 amino acid sequences were omitted. The sequence carries a GS deletion at this LC sequence that was used in the milk expression of the IgA1 version of the ZAC3 antibody. Constructs were completed which contained the LC (BC3236), J chain (BC2635), both the LC and J chain (BC3239), and the HC (BC3242) of IgA1. These constructs are listed in Supplemental Table 3 and described in detail in Supplemental Figures 1–4. We noted after the fact that the LC sequence in BC3239 is missing 2 codons (and therefore amino acids) from the original ZAC-3 LC, although functional studies indicate that the deletion did not significantly impact ZAC-3 functionality.

### Construction and production of ZAC-3 IgA1 milk-specific vectors and expression in mice

**Supplementary Figure 5.**
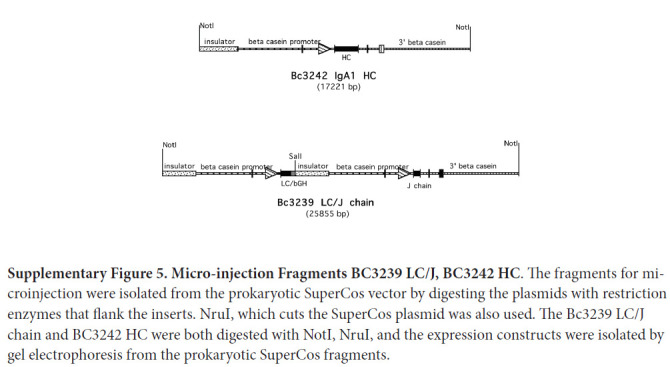
**Micro-injection Fragments BC3239 LC/J, BC3242 HC**. The fragments for microinjection were isolated from the prokaryotic SuperCos vector by digesting the plasmids with restriction enzymes that flank the inserts. NruI, which cuts the SuperCos plasmid was also used. The Bc3239 LC/J chain and BC3242 HC were both digested with NotI, NruI, and the expression constructs were isolated by gel electrophoresis from the prokaryotic SuperCos fragments.

**Supplementary Figure 6.**
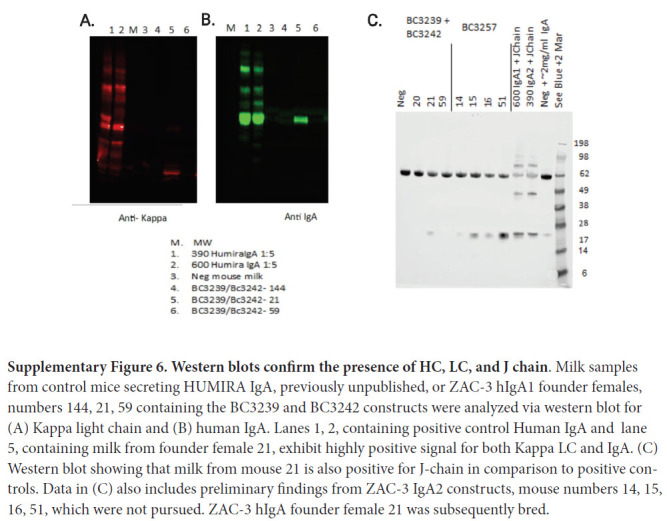
**Western blots confirm the presence of HC, LC, and J chain**. Milk samples from control mice secreting HUMIRA IgA, previously unpublished, or ZAC-3 hIgA1 founder females, numbers 144, 21, 59 containing the BC3239 and BC3242 constructs were analyzed via western blot for (A) Kappa light chain and (B) human IgA. Lanes 1, 2, containing positive control Human IgA and lane 5, containing milk from founder female 21, exhibit highly positive signal for both Kappa LC and IgA. (C) Western blot showing that milk from mouse 21 is also positive for J-chain in comparison to positive controls. Data in (C) also includes preliminary findings from ZAC-3 IgA2 constructs, mouse numbers 14, 15, 16, 51, which were not pursued. ZAC-3 hIgA founder female 21 was subsequently bred.

Transgenic mice were generated using BC3239 and BC3242 plasmids. The electroeluted fragments containing the eukaryotic sequences were mixed and micro-injected at 1ng/uL using standard procedures (Supplementary Figure 5). The progeny produced were analyzed for the presence of the transgenes by Transnetyx (https://www.transnetyx.com Transnetyx Inc. Cordova, TN). The samples were analyzed by qPCR using primers specific for J chain, LC, and HC (Supplementary Table 3). Twelve mice were found to be transgenic for all 3 genes. The 6 founder females were grown to maturity, bred, and brought into lactation. Their milk was analyzed for the presence of the MAbs by western blot. One line, No.21 produced relatively higher levels of the antibody and was chosen for strain expansion (Supplementary Figure 6). From this founder mouse are the F1, No.172 and 3 F2 females No.191, 192, 196. The m137 mouse did not produce antibody, so it was used as a negative control in the following experiments.

### Western blotting

Western blots were performed using goat anti-human IgA (AB_218398), with secondary rabbit anti-goat conjugated to Alexa Fluor 546 (AB_2535742), and anti-human J chain (AB_2121629) with LI-COR Biosciences anti-rabbit conjugated to IRDye 680RD secondary antibody (AB_10956166). Results confirm the presence of the HC (Supplementary Figure 6A), LC (Supplementary Figure 6B), and J chain (Supplementary Figure 6C).

### Bacterial agglutination assays

Agglutination assays were performed as described at the following link 10.17504/protocols.io.baahiab6 [23].

### ELISAs

Variations on the ELISA plate set up and antibodies used are described below. All ELISAs utilized Immunolon™ 4HBX 96-well microtiter ELISA plates and were developed using SureBlue™ Microwell Peroxidase Substrate. Plates were analyzed using a Spectromax 250 spectrophotometer with Softmax Pro 5.0 software (Molecular Devices).

Whole bacteria ELISAs were performed as previously described at the following link 10.17504/protocols.io.baajiacn [34].

An IgA sandwich ELISA was utilized to determine IgA concentration in milk. Plates were coated overnight with 1µg/mL of unlabeled Goat anti-human IgA (a-chain specific). Milk samples were serially diluted across plates, with human IgA from colostrum (Sigma-Aldrich Cat. No. I2636, RRID:AB_1163623) used as a control. A goat anti-human serum IgA conjugated to HRP secondary antibody was added at a 1:5,000 dilution to all wells (MP Biomedicals). For experiments testing the IgA concentration in the stomach contents of pups, we utilized this same assay, with the stomach contents mixed with protease inhibitor cocktail, described below, as the primary antibody.

For ZAC-3 IgG competition ELISAs, plates were coated with whole *V. cholerae* O395 cells as described above. All wells were subsequently coated with 0.1µg/mL of ZAC-3 IgG for 1 hour at room temperature. Plates were washed and IgA1-milk samples were incubated for 1 hour. A goat anti-human IgG conjugated to HRP secondary antibody was used at a 1:5,000 dilution for 1 hour (RRID:AB_2535582).

### Bacterial motility assays

Liquid motility and semi-solid agar (ssAgar) assays were performed as described previously [23].

### Animal care and ethics statement

The mouse experiments described in this study were reviewed and approved by the Wadsworth Center's Institutional Animal Care and Use Committee (IACUC) under protocol #17-428. The Wadsworth Center complies with the Public Health Service Policy on Humane Care and Use of Laboratory Animals and was issued assurance number A3183-01. The Wadsworth Center is fully accredited by the Association for Assessment and Accreditation of Laboratory Animal Care (AAALAC). Obtaining this voluntary accreditation status reflects that Wadsworth Center's Animal Care and Use Program meets all standards required by law and goes beyond these standards as it strives to achieve excellence in animal care and use. Mice were euthanized by carbon dioxide asphyxiation followed by cervical dislocation, as recommended by the Office of Laboratory Animal Welfare (OLAW), National Institutes of Health.

### Neonatal mouse model of cholera.

Neonatal mouse colonization studies were performed as described [22]. Two iterations of this experiment were done. For passive protection studies, 4- to 5-day-old BALB/c mice were removed from their dams and gavaged with 50 µL of ~1 × 10^7^ mid-log phase *V. cholerae* cells combined with the indicated milk treatment and blue food dye, utilizing a 24-gauge feeding needle (Harvard Apparatus; Cat. No. 75-0280). For experiments utilizing pups fed by IgA1 producing dams, pups were fed from control or ZAC-3 hIgA1 producing dams and gavaged with *V. cholerae* mixed with blue food dye only. At the time of gavage, 2 pups from every litter were euthanized, stomach and intestines taken, and homogenized as described below in 250 µL of PBS containing cOmplete, Mini, EDTA-free Protease Inhibitor Cocktail Tablets (Sigma Aldrich, Cat. No. 11836170001). Stomach and Intestinal contents from these pups were utilized in subsequent ELISAs and motility assays, as described previously. For all experiments gavaged pups were kept at 30^°^ C for 24 hours, euthanized via decapitation with sharp scissors, and whole intestines homogenized in 2 mL non-reinforced tubes (Fisher Scientific, Cat. No. 15-340-161) containing 4 to 5 UV-sterilized zirconium ceramic oxide beads (Fisher Scientific, Cat. No. 15-340-160) and 1mL of PBS. Intestines were homogenized utilizing a Fisherbrand™ Bead Mill 4 Homogenizer (Fisher Scientific, Cat. No. 15-340-164), run at 3 m/s for 30 seconds, 3 times. Homogenized intestines were plated to assess CFUs on indicated media.

## RESULTS

### Construction of a transgenic mouse expressing functional ZAC-3 hIgA1

The HC and LC variable sequences of ZAC-3 were cloned onto human IgA1 and κ chain expression vectors, respectively. The BC3239 construct contains the LC and J chain sequence and the BC3242 construct contains the HC sequence (Supplementary Figures 1–4). The ZAC-3 HC and LC and J chain sequences are under the control of a β-casein expression cassette, which includes the promoter and downstream untranslated region of the goat β-casein gene. The β-casein promoter is activated specifically in mammary epithelial cells during lactation, due to tissue-specific transcription factors and lactation-related hormonal requirements [35, 36]. The casein hIgA1 sequences were separated from prokaryotic sequencing following cleavage via restriction endonuclease digestion from the BC3239 and BC3242 constructs. They were introduced into mouse embryos via microinjection (Supplementary Figure 5) [37]. Candidate transgenic mice were tested by PCR for the presence of both the LC-J chain cassette (BC3239) and the HC cassette (BC3242) using primers listed in Supplementary Table 3.

**Figure 1.**
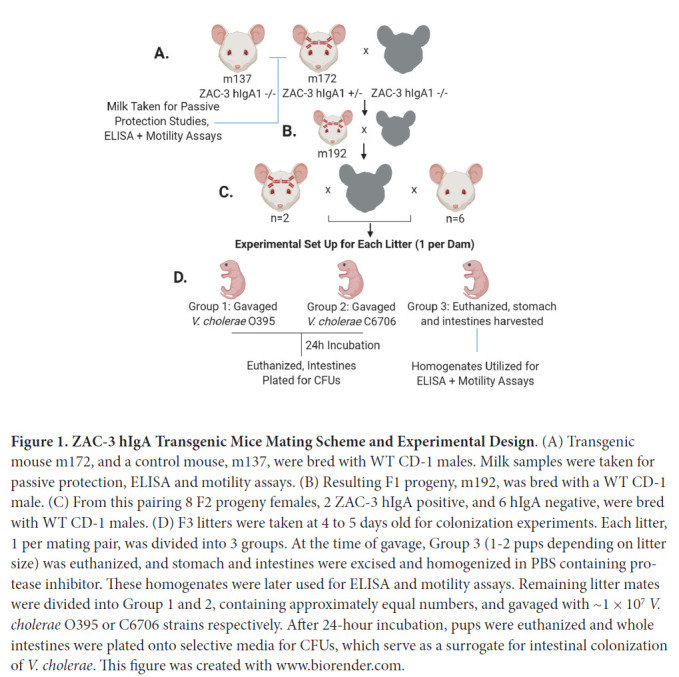
**ZAC-3 hIgA Transgenic Mice Mating Scheme and Experimental Design**. (A) Transgenic mouse m172, and a control mouse, m137, were bred with WT CD-1 males. Milk samples were taken for passive protection, ELISA and motility assays. (B) Resulting F1 progeny, m192, was bred with a WT CD-1 male. (C) From this pairing 8 F2 progeny females, 2 ZAC-3 hIgA positive, and 6 hIgA negative, were bred with WT CD-1 males. (D) F3 litters were taken at 4 to 5 days old for colonization experiments. Each litter, 1 per mating pair, was divided into 3 groups. At the time of gavage, Group 3 (1-2 pups depending on litter size) was euthanized, and stomach and intestines were excised and homogenized in PBS containing protease inhibitor. These homogenates were later used for ELISA and motility assays. Remaining litter mates were divided into Group 1 and 2, containing approximately equal numbers, and gavaged with ~1 × 10^7^ *V. cholerae* O395 or C6706 strains respectively. After 24-hour incubation, pups were euthanized and whole intestines were plated onto selective media for CFUs, which serve as a surrogate for intestinal colonization of *V. cholerae*. This figure was created with www.biorender.com.

**Figure 2.**
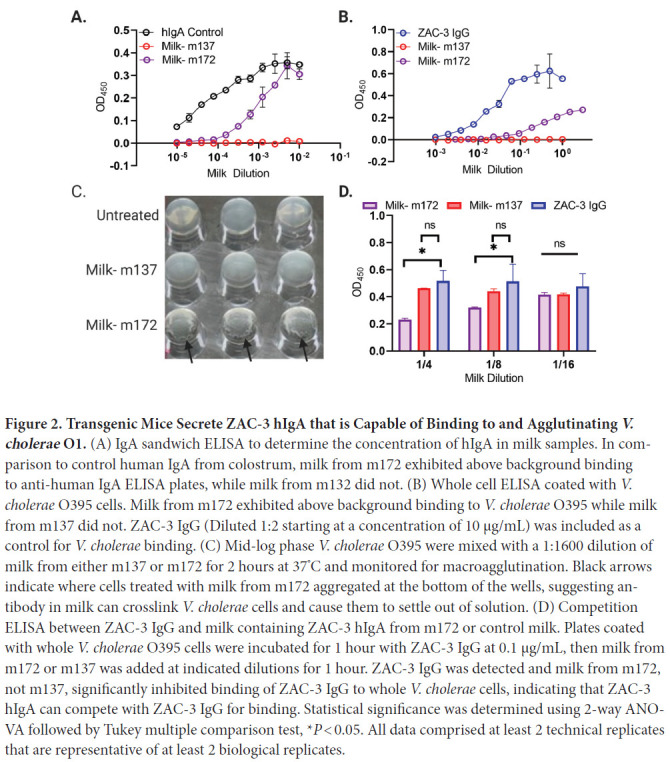
**Transgenic Mice Secrete ZAC-3 hIgA that is Capable of Binding to and Agglutinating *V. cholerae* O1.** (A) IgA sandwich ELISA to determine the concentration of hIgA in milk samples. In comparison to control human IgA from colostrum, milk from m172 exhibited above background binding to anti-human IgA ELISA plates, while milk from m132 did not. (B) Whole cell ELISA coated with *V. cholerae* O395 cells. Milk from m172 exhibited above background binding to *V. cholerae* O395 while milk from m137 did not. ZAC-3 IgG (Diluted 1:2 starting at a concentration of 10 µg/mL) was included as a control for *V. cholerae* binding. (C) Mid-log phase *V. cholerae* O395 were mixed with a 1:1600 dilution of milk from either m137 or m172 for 2 hours at 37^°^C and monitored for macroagglutination. Black arrows indicate where cells treated with milk from m172 aggregated at the bottom of the wells, suggesting antibody in milk can crosslink *V. cholerae* cells and cause them to settle out of solution. (D) Competition ELISA between ZAC-3 IgG and milk containing ZAC-3 hIgA from m172 or control milk. Plates coated with whole *V. cholerae* O395 cells were incubated for 1 hour with ZAC-3 IgG at 0.1 µg/mL, then milk from m172 or m137 was added at indicated dilutions for 1 hour. ZAC-3 IgG was detected and milk from m172, not m137, significantly inhibited binding of ZAC-3 IgG to whole *V. cholerae* cells, indicating that ZAC-3 hIgA can compete with ZAC-3 IgG for binding. Statistical significance was determined using 2-way ANOVA followed by Tukey multiple comparison test, *P < 0.05. All data comprised at least 2 technical replicates that are representative of at least 2 biological replicates.

Milk was collected from 2 PCR-confirmed transgenic mice (m137 and m172) and tested for the presence of human IgA by western blot and in a human IgA-specific sandwich ELISA (see Materials and Methods). The mouse m172 was an F1 progeny of the expressing animal m21, shown in the western blot (Supplementary Figure 6). An overview of mating and experimental workflow is shown in Figure 1. Milk from m172 had 300 to 600 µg/mL of human IgA (Figure 2A), while m137 showed no detectable reactivity. Milk from m172 bound *V. cholerae* O1 classical Ogawa strain O395 by whole cell ELISA, demonstrating the presence of functional ZAC-3 IgA in milk (Figure 2B). We utilized a competition ELISA to further assess if ZAC-3 IgA1 retained the same epitope specificity as the parental ZAC-3 IgG construct. ZAC-3 hIgA1 milk samples significantly inhibited chimeric ZAC-3 IgG from binding to whole *V. cholerae* O395 cells (Figure 2D), indicating that transgenic CD-1 mice secrete human ZAC-3 hIgA1 that retains the core/lipid A epitope specificity. Finally, milk from m172 (but not 137) promoted *V. cholerae* O395 agglutination in vitro even at >1:1600 dilution (Figure 2C). Of note, ZAC-3 hIgA1 antibody was secreted by mammary epithelial cells, not secreted across the epithelial layer. As such the secreted product is dimeric (dIgA) but not necessarily complexed with secretory component to form secretory IgA (SIgA).

### Passive immunity afforded by milk-derived ZAC-3 hIgA1

**Supplementary Figure 7.**
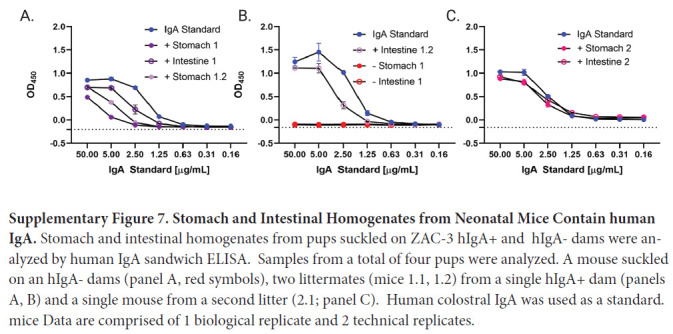
**Stomach and Intestinal Homogenates from Neonatal Mice Contain human IgA.** Stomach and intestinal homogenates from pups suckled on ZAC-3 hIgA+ and hIgA- dams were analyzed by human IgA sandwich ELISA. Samples from a total of four pups were analyzed. A mouse suckled on an hIgA- dams (panel A, red symbols), two littermates (mice 1.1, 1.2) from a single hIgA+ dam (panels A, B) and a single mouse from a second litter (2.1; panel C). Human colostral IgA was used as a standard. mice Data are comprised of 1 biological replicate and 2 technical replicates.

**Supplementary Figure 8.**
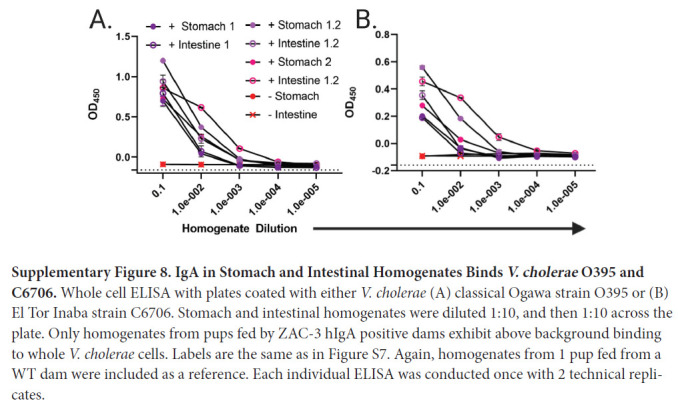
**IgA in Stomach and Intestinal Homogenates Binds *V. cholerae* O395 and C6706.** Whole cell ELISA with plates coated with either *V. cholerae* (A) classical Ogawa strain O395 or (B) El Tor Inaba strain C6706. Stomach and intestinal homogenates were diluted 1:10, and then 1:10 across the plate. Only homogenates from pups fed by ZAC-3 hIgA positive dams exhibit above background binding to whole *V. cholerae* cells. Labels are the same as in Figure S7. Again, homogenates from 1 pup fed from a WT dam were included as a reference. Each individual ELISA was conducted once with 2 technical replicates.

To test if milk-expressed ZAC-3 hIgA1 affects bacterial colonization of the intestinal epithelium, we utilized the neonatal mouse model of cholera colonization [38]. CD-1 pups (4-5 days old) nursed by control dams or dams producing ZAC-3 hIgA1 were gavaged with either *V. cholerae* classical Ogawa O395 or El Tor Inaba strain C6706 cells. Stomach and intestinal contents from pups were taken at the time of gavage and tested for the presence of human IgA via ELISA to quantitate human IgA levels at the time of challenge. As determined by IgA sandwich ELISA, the stomachs and intestines isolated from pups in the ZAC-3 hIgA-positive groups contained approximately 20 to 40 µg/ml human IgA, while hIgA-negative groups had no detectable human IgA (Supplementary Figure 7). The stomach and intestinal homogenates from the ZAC-3 hIgA-positive groups bound whole *V. cholerae* O1 classical Ogawa strain O395 and El Tor Inaba strain C6706 by whole cell ELISA, demonstrating that ZAC-3 IgA taken in by nursing pups retains functionality and reactivity with both serotypes within the *V. cholerae* O1 serogroup (Supplementary Figure 8).

**Figure 3.**
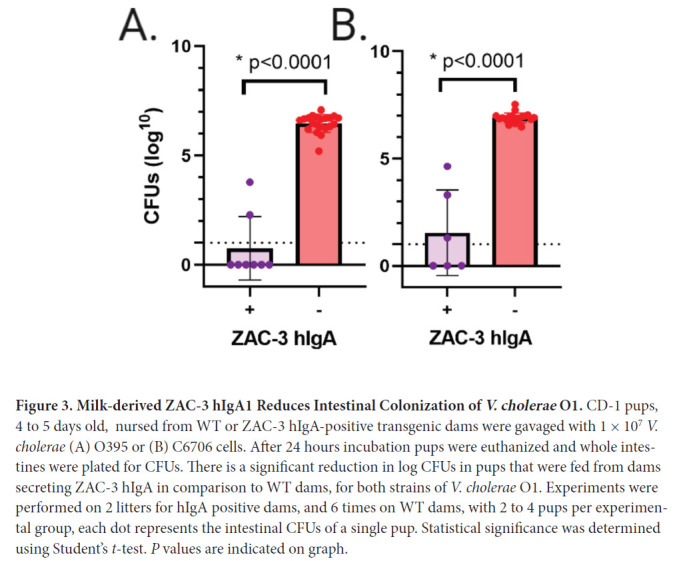
**Milk-derived ZAC-3 hIgA1 Reduces Intestinal Colonization of *V. cholerae* O1.** CD-1 pups, 4 to 5 days old, nursed from WT or ZAC-3 hIgA-positive transgenic dams were gavaged with 1 × 10^7^ *V. cholerae* (A) O395 or (B) C6706 cells. After 24 hours incubation pups were euthanized and whole intestines were plated for CFUs. There is a significant reduction in log CFUs in pups that were fed from dams secreting ZAC-3 hIgA in comparison to WT dams, for both strains of *V. cholerae* O1. Experiments were performed on 2 litters for hIgA positive dams, and 6 times on WT dams, with 2 to 4 pups per experimental group, each dot represents the intestinal CFUs of a single pup. Statistical significance was determined using Student's *t*-test. *P* values are indicated on graph.

Eight litters of pups were gavaged with *V. cholerae* strains O395 or C6706. Among these litters, 2 were ZAC-3 hIgA positive and 6 were hIgA negative. Pups gavaged with *V. cholerae* strains O395 or C6706 were euthanized after 24 hours, at which time intestines were excised, homogenized, and plated on LB agar to enumerate *V. cholerae* CFUs as a surrogate for colonization. Pups gavaged with *V. cholerae* strains O395 or C6706 in the ZAC-3 hIgA-positive group showed a signifi-cant reduction in colonization in both strains in comparison to the hIgA-negative groups (Figure 3).

**Figure 4.**
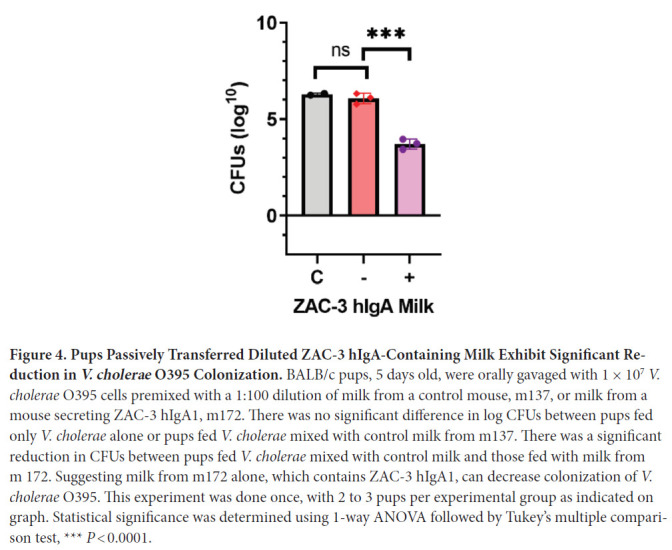
**Pups Passively Transferred Diluted ZAC-3 hIgA-Containing Milk Exhibit Significant Reduction in *V. cholerae* O395 Colonization.** BALB/c pups, 5 days old, were orally gavaged with 1 × 10^7^ *V. cholerae* O395 cells premixed with a 1:100 dilution of milk from a control mouse, m137, or milk from a mouse secreting ZAC-3 hIgA1, m172. There was no significant difference in log CFUs between pups fed only *V. cholerae* alone or pups fed *V. cholerae* mixed with control milk from m137. There was a significant reduction in CFUs between pups fed *V. cholerae* mixed with control milk and those fed with milk from m 172. Suggesting milk from m172 alone, which contains ZAC-3 hIgA1, can decrease colonization of *V. cholerae* O395. This experiment was done once, with 2 to 3 pups per experimental group as indicated on graph. Statistical significance was determined using 1-way ANOVA followed by Tukey's multiple comparison test, *** *P* < 0.0001.

We next performed passive transfer studies to ensure that the milk from the ZAC-3 hIgA1 transgenic dams, and not another variable, was responsible for protection against *V. cholerae* colonization. ZAC-3 hIgA1-containing milk, or control milk was diluted 1:100 (3-6 µg/mL), mixed with *V. cholerae* O395 and administered to 4-day-old BALB/c pups. Pups treated with ZAC-3 hI-gA1-containing milk exhibited a 2-log reduction of CFUs in intestinal homogenates compared to pups treated with control milk (Figure 4). Together these data show that pups fed from transgenic ZAC-3 hIgA1 dams contain milk that is positive for hIgA and exhibit decreased colonization in comparison to pups fed from hIgA-negative dams.

### Effect of ZAC-3 hIgA1 on *V. cholerae* motility

**Figure 5.**
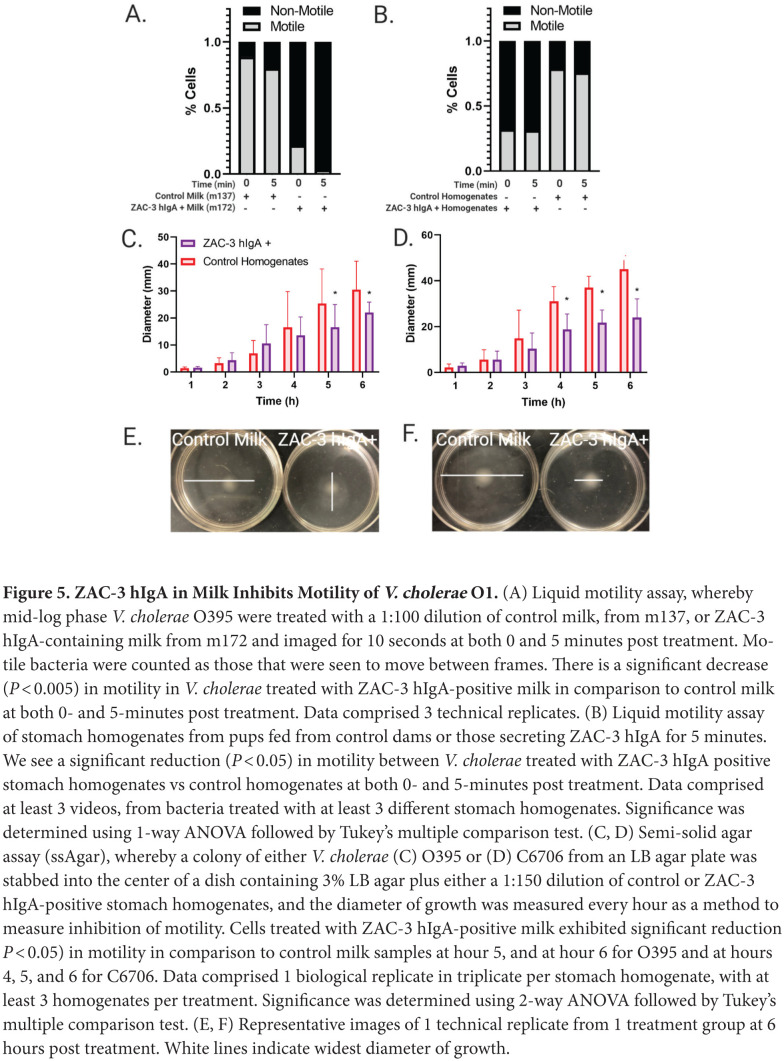
**ZAC-3 hIgA in Milk Inhibits Motility of *V. cholerae* O1.** (A) Liquid motility assay, whereby mid-log phase *V. cholerae* O395 were treated with a 1:100 dilution of control milk, from m137, or ZAC-3 hIgA-containing milk from m172 and imaged for 10 seconds at both 0 and 5 minutes post treatment. Motile bacteria were counted as those that were seen to move between frames. There is a significant decrease (*P* < 0.005) in motility in *V. cholerae* treated with ZAC-3 hIgA-positive milk in comparison to control milk at both 0- and 5-minutes post treatment. Data comprised 3 technical replicates. (B) Liquid motility assay was performed as described, with *V. cholerae* O395 expressing mCherry treated with either a 1:100 dilution of stomach homogenates from pups fed from control dams or those secreting ZAC-3 hIgA for 5 minutes. We see a significant reduction (*P* < 0.05) in motility between *V. cholerae* treated with ZAC-3 hIgA positive stomach homogenates vs control homogenates at both 0- and 5-minutes post treatment. Data comprised at least 3 videos, from bacteria treated with at least 3 different stomach homogenates. Significance was determined using 1-way ANOVA followed by Tukey's multiple comparison test. (C, D) Semi-solid agar assay (ssAgar), whereby a colony of either *V. cholerae* (C) O395 or (D) C6706 from an LB agar plate was stabbed into the center of a dish containing 3% LB agar plus either a 1:150 dilution of control or ZAC-3 hIgA-positive stomach homogenates, and the diameter of growth was measured every hour as a method to measure inhibition of motility. Cells treated with ZAC-3 hIgA-positive milk exhibited significant reduction *P* < 0.05) in motility in comparison to control milk samples at hour 5, and at hour 6 for O395 and at hours 4, 5, and 6 for C6706. Data comprised 1 biological replicate in triplicate per stomach homogenate, with at least 3 homogenates per treatment. Significance was determined using 2-way ANOVA followed by Tukey's multiple comparison test. (E, F) Representative images of 1 technical replicate from 1 treatment group at 6 hours post treatment. White lines indicate widest diameter of growth.

The effect of anti-LPS IgA antibodies on *V. cholerae* colonization in the mouse model has been attributed to inhibition of flagellar motility [18, 22, 24]. We therefore tested the impact of milk containing ZAC-3 hIgA1 on *V. cholerae* motility in a liquid motility assay. *V. cholerae* O395 cells were treated with milk from mouse 137 (negative) or mouse 172 (ZAC-3 hIgA1 positive), in liquid LB for 5 minutes, and 10s videos were captured at the 0 and 5-minute post-treatment time points. *V. cholerae* O395 cells treated with milk containing ZAC-3 hIgA1 exhibited significant decrease in motility over the course of 5 minutes (Figure 5A; Supplementary Videos 1 and 2).

To support these results, we utilized the liquid motility assay on stomach contents from the hIgA-containing milk fed to pups, as described previously. The stomach contents from pups fed from either ZAC-3 hIgA-positive or negative dams were diluted 1:100 into LB containing midlog phase *V. cholerae* O395 expressing mCherry on a plasmid. We saw a reduction in motility in bacteria that were treated with stomach contents from the ZAC-3 hIgA-positive pups, in comparison to the control samples (Figure 5B, Supplementary Video 3 and 4). We then utilized the ssAgar assay, whereby stomach contents from ZAC-3 hIgA1 or controls were diluted 1:150 into 0.3% LB agar, and colonies of *V. cholerae* O395 and C6706 were stabbed into the agar. Diameter of growth was measured over the course of 6 hours to measure the inhibition of motility. We showed that bacteria treated with stomach contents from the ZAC-3 hIgA-positive group exhibited decreased motility in comparison to milk from the control groups over the course of the 6-hour assay (Figure 5C-F). Collectively this demonstrates that milk from ZAC-3 hIgA1 transgenic animals can significantly inhibit bacterial motility.

**Supplementary Figure 9.**
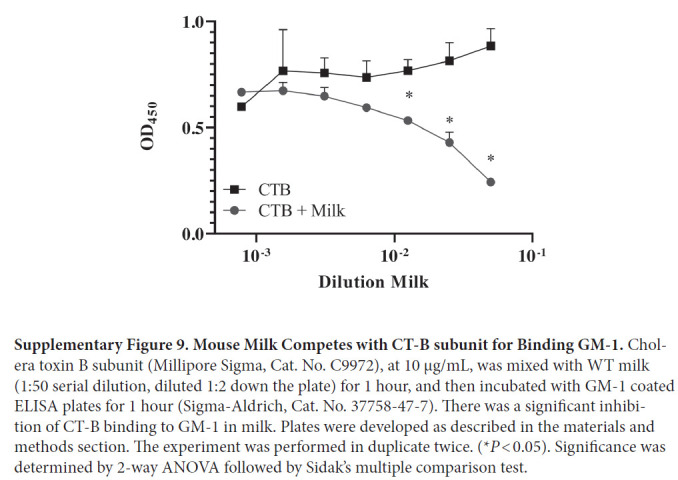
**Mouse Milk Competes with CT-B subunit for Binding GM-1.** Cholera toxin B subunit (Millipore Sigma, Cat. No. C9972), at 10 µg/mL, was mixed with WT milk (1:50 serial dilution, diluted 1:2 down the plate) for 1 hour, and then incubated with GM-1 coated ELISA plates for 1 hour (Sigma-Aldrich, Cat. No. 37758-47-7). There was a significant inhibition of CT-B binding to GM-1 in milk. Plates were developed as described in the materials and methods section. The experiment was performed in duplicate twice. (*P < 0.05). Significance was determined by 2-way ANOVA followed by Sidak's multiple comparison test.

Lastly, it should be noted that 1 benefit of the milk-based expression system is that the MAbs are delivered along with other natural components of milk, including lactoferrin, secretory component and so on [39]. In the case of cholera, it has been reported that milk itself is able to inhibit CT binding to ganglioside (GM-1) on intestinal epithelial cells [40, 41]. Indeed, we confirmed this observation in our model (Supplementary Figure 9). This is a potential added benefit of milk-based production and delivery of antibody therapy.

## DISCUSSION

The current study represents a first step towards the prospect of developing a passive MAb-based oral immunization regimen as a supplement to OCV strategies, particularly in outbreak situations where cholera incidence can outpace vaccine-induced immunity. The 2 WHO pre-qualified OCVs, for example, are administered as 2 or 3 doses at approximately 2-week intervals with overall protective efficacy ranging from 60% to 85% [42, 43]. In outbreak situations, a self-administrable, pre-exposure prophylactic could be dispensed to at-risk individuals during the days leading up to or in between OCV dosing. Theoretically, such an intervention could be implemented at the community and household levels to avert disease in particularly high-risk individuals, especially in close contact situations [44].

Our study confirms, in a mouse model, that passive administration of anti-LPS IgA antibodies has significant benefit in terms of reducing intestinal colonization of *V. cholerae*. Anti-LPS antibodies can be either polyclonal or monoclonal in nature. For example, Bishop and colleagues demonstrated in the neonatal mouse model that protection (ie, reduced colonization) was observed when pups were suckled on dams previously vaccinated with *V. cholerae* outer membrane vesicle preparations. Immunity was associated with anti-LPS antibody titers (predominantly IgG and secondarily IgA) in milk, which in turn correlated with the ability of immune milk to arrest bacterial motility [18]. Prior to that study, Winner and colleagues demonstrated in the so-called backpack tumor model that a single IgA MAb directed against Owaga-specific epitope was protective against a lethal cholera challenge [17]. There is also evidence to suggest a benefit of passively administered anti-LPS IgA in preventing cholera in humans. Epidemiological evidence from Bangladesh reveals that the incidence of cholera is lower in breast fed children, and that the active factor in milk is primarily anti-LPS IgA [28].

ZAC-3, which was originally isolated from a mouse Peyer's patch-derived B-cell hybridoma, is unusual in that it is directed against an epitope within the core/lipid A region of *V. cholerae* O1 LPS shared across all clinical isolates we have tested [30, 31]. In humans (and mice for that matter), most anti-*V. cholerae* LPS antibodies target OPS [11]. However, the atypical nature of ZAC-3 should not exclude it from consideration for development as a putative prophylactic, especially considering the fact that its epitope is conserved across *V. cholerae* O1 serotypes [30, 32]. ZAC-3 IgG has been shown to inhibit flagella-based motility and agglutinates Classical and El Tor clinical isolates, 2 effector functions that are thought to contribute to immunity in animal models [32]. In this study, passively transferred milk containing ZAC-3 hIgA1 diluted 1:100 premixed with *V. cholerae* resulted in a ~2-log reduction in CFUs recovered from neonatal mouse intestines (Figure 4). In human clinical trials it has been demonstrated that even small changes in challenge dose (+/- 1 log CFU) can have a significant impact on clinical presentation (eg, asymptomatic vs symptomatic) [45]. Therefore, it is likely that use of ZAC-3 hIgA1-containing milk as a prophylactic MAb therapy would result in a significant difference in clinical outcome in comparable human trials. In terms of scale-up and production, it has already been shown that ZAC-3 retains its biological efficacy when expressed as a recombinant human IgG1 in a *Nicotiana*-based platform [22, 34] and now as a human dimeric IgA1 molecule in a mammalian mammary gland system.

One caveat of our current study is that ZAC-3 IgA was expressed as a dimeric IgA1 molecule without the addition of secretory component (SC). The SC is a ~75 kDa glycoprotein derived from the pIgR that normally covalently associates with dimeric IgA during transcytosis across mucosal epithelia, including the mammary epithelium [46]. In the current model, ZAC-3 IgA was expressed by mammary epithelial cells (under control of a β-casein expression cassette) and secreted directly from the cells, independent of pIgR-mediated transport. Nonetheless, there is a small amount of free SC in breast milk, so it is conceivable that a fraction of ZAC-3 dimeric IgA1 did in fact convert to SIgA. We would expect that ZAC-3 SIgA would have additional benefits in terms of protective immunity, as SC imparts a number of unique traits upon IgA, including improved GI stability and association with mucus. SIgA may also synergize with other factors in breast milk, including lactoferrin. Finally, there are reports that human SC specifically affects *V. cholerae* biofilm formation and therefore might affect intestinal colonization [47]. It remains to be tested whether ZAC-3 SIgA would have had benefits greater than IgA alone.

In summary, we show here the effectiveness of the milk-expression platform in producing a previously characterized monoclonal antibody directed against a cross-protective epitope on *V. cholerae* O1 LPS. This work supports the milk-production platform as a viable mechanism of producing prodigious amounts of IgA, with real-world application for utilization of this platform for production in larger animals, ie, cows and goats, in order to combat many diseases of public health importance in high-risk populations.

## Figures and Tables

**Supplementary Video 1.** ***V. cholerae* O1 strain O395 Remains Motile When Treated with Control Milk.** Mid-log phase *V. cholerae* O395 in LB were treated with a 1:100 dilution of control milk, from mouse 137. Video is a compilation of 10s videos, 100 total frames per video, played at 10 frames per second, captured at time 0, and 5 minutes post treatment. Bacteria treated with control milk remain motile throughout the 5-minute time period, as evidenced by swirling bacteria coming into and out of focus. Video is representative of 3 technical replicates.

**Supplementary Video 2.** ***V. cholerae* O1 strain O395 Is Rendered Immobile When Treated with Milk Containing ZAC-3 hIgA1.** Mid-log phase *V. cholerae* O395 in LB were treated with a 1:100 dilution of milk containing ZAC-3 hIgA, from mouse 172. Video is a compilation of 10s videos, 100 total frames per video, played at 10 frames per second, captured at time 0, and 5 minutes post treatment. Bacteria treated with ZAC-3 hIgA-containing milk do not exhibit flagellar-based motility throughout the 5-minute time period, as evidenced by lack of swirling bacteria coming into and out of focus. Video is representative of 3 technical replicates.

**Supplementary Video 3.** ***V. cholerae* O1 strain O395 Remains Motile When Treated with Stomach Homogenates from Control Pups.** Mid-log phase *V. cholerae* O395 expressing a plasmid with mCherry in LB were treated with a 1:100 dilution of stomach homogenates from a 4- to 5-day-old pup nursed from a control, WT CD-1 dam. Video is a compilation of 10s videos, 10 total frames per video, played at 10 frames per second, captured at time 0, and 5 minutes post treatment. Bacteria treated with control homogenates remain motile throughout the 5-minute time period, as evidenced by swirling bacteria coming into and out of focus. Video is representative of 11 individual experiments with stomach homogenates from pups fed from 6 WT CD-1 dams.

**Supplementary Video 4.** ***V. cholerae* O1 strain O395 Is Rendered Immobile When Treated with Stomach Homogenates Containing ZAC-3 hIgA1.** Mid-log phase *V. cholerae* O395 expressing a plasmid with mCherry in LB were treated with a 1:100 dilution of stomach homogenates from a 4- to 5-day-old pup nursed from a transgenic CD-1 dam that secretes ZAC-3 hIgA in milk. Video is a compilation of 10s videos, 10 total frames per video, played at 10 frames per second, captured at time 0, and 5 minutes post treatment. Bacteria treated with ZAC-3 hIgA-containing homogenates exhibit less flagellar-based motility throughout the 5-minute experiment, as shown by quantitation of the number of moving bacteria in Figure 5B. Video is representative of at least 3 individual experiments with stomach homogenates from pups fed from at least 2 ZAC-3 hIgA1 CD-1 dams.

## SUPPLEMENTARY MATERIALS

### Supplementary Videos Are Provided Via the Following Links

https://www.youtube.com/watch?v=W6TtMeEf5xA

https://www.youtube.com/watch?v=OddO2TXc8SA

https://www.youtube.com/watch?v=V-k0fKW735U

https://www.youtube.com/watch?v=s1PLyMxtOUM

### SUPPLEMENTARY TABLES

**Supplementary Table 1.** List of bacterial strains utilized in this work.

Strain/Name	Characteristics/Sequence	Source/Reference
*V. cholerae* O395	Wild-type Classical Ogawa	John Mekalanos (Harvard Medical School)
*V. cholerae* C6706	Wild-type El Tor Inaba	Christopher Waters (Michigan State Univ.)
DB364	*V. cholerae* O395 pGW104	*This work*

**Supplementary Table 2.** List of primers utilized in this work.

Name	Sequence	Source/Reference
BC3242 FWD	CCGTGACTTGGAGCGAATCT	*This work*
BC3242 REV	GCGTCCTGAGAAGGTGGG	*This work*
BC3239 FWD	GACCTGGCCGAGTACTTCTG	*This work*
BC3239 REV	GTCCTCTTGATTTCCAGCTTGGT	*This work*
PA1/04/03_F_Kpn1	ATA GGT ACC ATT TAT CAG GGT TAT TGT CTC	*This work*
	ATG A	
PA1/04/03_ORF_SOE_R1	CCT TGC TCA CCA TGC TTA ATT TCT CCT CTT TAA TTC TAG ATG TG	*This work*
xFP_ORF_SOE_F2	ATT AAA GAG GAG AAA TTA AGC ATG GTG AGC AAG GGC GAG GAG	*This work*
mcherry_R_hindIII	TTA AAG CTT GCA TGC CTG CAG ACT AGT CTA CT	*This work*

**Supplementary Table 3.** List of plasmids utilized in this work.

Plasmid Name	Characteristics/Sequence	Source/Reference
pBC1	Contains goat β-casein expression cassette	Invitrogen
BC451	Beta Casein expression vector encoding a 7.2kb downstream sequence including the last 3 exons of beta casein	(X. Yu *et al.* 2013)
BC350	Beta Casein expression vector encoding a 7.2kb downstream sequence including the last 3 exons of beta casein	(X. Yu *et al.* 2013)
BC2797	Beta Casein expression vector encoding a 300bp bgH polyA sequence	(X. Yu *et al.* 2013)
BC3242	BC451 encoding ZAC-3 IgA1 HC (**Figure S1**)	*This work*
BC3235	BC2797 encoding ZAC-3 IgA1 LC (**Figure S2**)	*This work*
BC2635	BC350 encoding J chain (**Figure S3**)	*This work*
BC3239	Notl-Sall fragment of BC3235 and Sall-NotI fragment of BC2635 ligated into Supercos (**Figure S4**)	*This work*
pMQCherry80	*mCherry* open reading frame	Matthew Wargo, unpublished
pUC18-mTn7T-eyfp-Gm	constitutive derivative of the *P_lac_* promoter (*P_A/01/04/03_*)	[33]
pGW104	pUC18T-mTn7T-eyfp with constitutive P_lac_ promotor and mCherry ORF	*This work*
